# An Overview of Commercially Available Apps in the Initial Months of the COVID-19 Pandemic

**DOI:** 10.3389/fpsyt.2021.557299

**Published:** 2021-04-15

**Authors:** Melvyn W. B. Zhang, Aloysius Chow, Roger C. M. Ho, Helen E. Smith

**Affiliations:** ^1^Family Medicine and Primary Care, Lee Kong Chian School of Medicine, Nanyang Technological University Singapore, Singapore, Singapore; ^2^National Addictions Management Service, Institute of Mental Health, Singapore, Singapore; ^3^Department of Psychological Medicine, Yong Loo Lin School of Medicine, National University of Singapore, Singapore, Singapore

**Keywords:** COVID-19, smartphone apps, M-health, technology, review

## Abstract

**Introduction:** It has been 4 months since the discovery of COVID-19, and there have been many measures introduced to curb movements of individuals to stem the spread. There has been an increase in the utilization of web-based technologies for counseling, and for supervision and training, and this has been carefully described in China. Several telehealth initiatives have been highlighted for Australian residents. Smartphone applications have previously been shown to be helpful in times of a crisis. Whilst there have been some examples of how web-based technologies have been used to support individuals who are concerned about or living with COVID-19, we know of no studies or review that have specifically looked at how M-Health technologies have been utilized for COVID-19.

**Objectives:** There might be existing commercially available applications on the commercial stores, or in the published literature. There remains a lack of understanding of the resources that are available, the functionality of these applications, and the evidence base of these applications. Given this, the objective of this content analytical review is in identifying the commercial applications that are available currently for COVID-19, and in exploring their functionalities.

**Methods:** A mobile application search application was used. The search terminologies used were “COVID” and “COVID-19.” Keyword search was performed based on the titles of the commercial applications. The search through the database was conducted from the 27th March through to the 18th of April 2020 by two independent authors.

**Results:** A total of 103 applications were identified from the Apple iTunes and Google Play store, respectively; 32 were available on both Apple and Google Play stores. The majority appeared on the commercial stores between March and April 2020, more than 2 months after the first discovery of COVID-19. Some of the common functionalities include the provision of news and information, contact tracking, and self-assessment or diagnosis.

**Conclusions:** This is the first review that has characterized the smartphone applications 4 months after the first discovery of COVID-19.

## Introduction

On the 31st of December 2019, the World Health Organization (WHO) was alerted by authorities in China of a case of pneumonia of unknown origin (1). Within weeks of the detection of the index case, large number of individuals were afflicted, and the total number of cases and mortality exceeded that of the Severe Acute Respiratory Syndrome (SARS) outbreak in 2003. On the 30th of January 2020, the WHO declared the outbreak as a public health emergency (1). The rapid increase in the number of cases and the increasing rates of mortality resulted in the WHO's declaration of the outbreak as a pandemic (COVID-19) on the 11th March 2020 (1). To date, as of the 21st of April 2020, a total of 2,314,621 cases have been confirmed globally, along with a total of 157,847 deaths (2). Throughout the world, countries are trying their best to contain this pandemic, with some countries, like Italy, Spain and Malaysia, locking down their cities; whilst others have adopted stringent measures such an increased social distancing in their attempt to stem the community spread of COVID-19 (3, 4).

Many of the measures introduced to stem the rapid community spread of COVID-19 involve individuals being confined to their homes, with only limited movement. In parallel there has been an increase in the utilization of web-based technologies for counseling, and for supervision and training, and this has been carefully described in China (5). The Chinese government has also tapped upon social media technologies, such as that of WeChat and Tencent QQ, to provide psychoeducation, which is much needed in times of crisis (5). Telehealth technologies have also been used to support frontline healthcare workers and patients who are diagnosed with COVID-19, and there is evidence demonstrating the feasibility and acceptability of such technologies (5). Most recently, Zhou et al. (6) have identified tele-mental health services available in Australia, that could potentially be used to meet the unprecedented need for mental health care and treatment. The existing tele-health initiatives in Australia are mostly delivered on online platforms, but there are also apps, such as that by Black Dog Institute to help individuals with mood and anxiety disorders (6).

Smartphone applications have previously been shown to be helpful in times of a crisis to assess the impact on psychological well-being. Zhang et al. (7) used social media and an accompanying smartphone application to assess for psychological distress amongst the general public during the 2013 Southeast Asia Haze Crisis. Algahtani et al. (8) described the use of the iPhone in delivering a questionnaire to assess public response to MERS-COV. Mobile applications, such as “Flu-Report” have been helpful in the acquisition of real-time information about the spread of influenza using self-reported information. Fujibayashi et al. (9). All these examples illustrate the great potential of mobile technologies during times of a crisis, including epidemics. Whilst there have been some examples of how web-based technologies have been used to support individuals who are concerned about or living with COVID-19, we know of no studies or review that have specifically looked at how M-Health technologies have been utilized for COVID-19, especially so in the initial months of the pandemic. There might be existing commercially available applications on the commercial stores, or in the published literature. There remains a lack of understanding of the resources that are available, the functionality of these applications, and the evidence base of these applications. It is of importance for there to be an understanding of the immediate tools that were made available to the public, as with the lockdowns and restrictions in movements, individuals could only obtain information digitally; and receive interventions digitally. Four months since the first index case of COVID-19 was detected in China, it is now timely to review the applications that have been designed to help reduce and alleviate the distress associated this global pandemic. Given this, the objective of this content analytical review is in identifying the commercial applications that are available currently for COVID-19, and in exploring their functionalities.

## Methods

### Identification of COVID-19 Applications on the Commercial Stores

The methodology we adopted for this review was based on that used previously to identify attention and cognitive bias modification applications (10). The previous review involved a manual cross-sectional search on the apps store iTunes (Apple Inc, Cupertino, CA, USA) and Google Play (Google LLC, Mountain View, CA, USA). On this occasion we dispensed with a manual search as we anticipated the yield would be low, given that manual searches are only able to identify applications from a particular country or locality (due to the restrictions on the app store in searching for apps that are available in other countries). Instead we used a mobile application search application, App Annie (11), this is a mobile application search engine that is freely available and equivalent to 42 matters (AG, Zurich, Switzerland), which we have used previously. App Annie is a database that collates commercial applications in various application stores, and provides analytics into the performance of these applications (i.e., uptake rates). The search terms used were “COVID” and “COVID-19.” Keyword search was performed based on the titles of the commercial applications. We did not limit the search of the applications by means of the language of the applications but included all applications that provided information of how they were used for COVID-19.

The search through the database was conducted from the 27th March through to the 18th of April 2020 by two independent authors (MZ and AC). We have decided to search for commercial applications during the acute/initial phase of the COVID-19 pandemic, in order to better understand how commercially available COVID-19 applications have helped in the initial stage of the pandemic. We concluded the search when there had been 3 consecutive days with no new countries or areas infected with COVID-19.Both reviewers independently identified the applications and compiled a list of those relevant. Applications were deemed to be relevant if they described how they provided some means of intervention for the COVID-19 pandemic.

In the event of any disagreement between the two reviewers this was resolved with discussion with the third researcher. An electronic spreadsheet was used in the collation of the following information from the identified applications, that of (a) Name of app, (b) App store notes (if the description were not in English, Google translate was used), (c) Functionality (based on the app store notes description), (d) country, (e) Category, (f) Release date, (g) Last updated date, (h) Version, (i) Seller, (j) App support link, (k) Privacy policy, (l) Displayed average ratings, and (m) Total displayed ratings.

As some of the identified applications were designed for use in non-English speaking countries Google translate was used for us to understand the description of the applications. We were not able to download the identified applications for a full assessment of their functionalities, given that we were unable to access applications published outside of Singapore.

## Results

**Figure 1:**
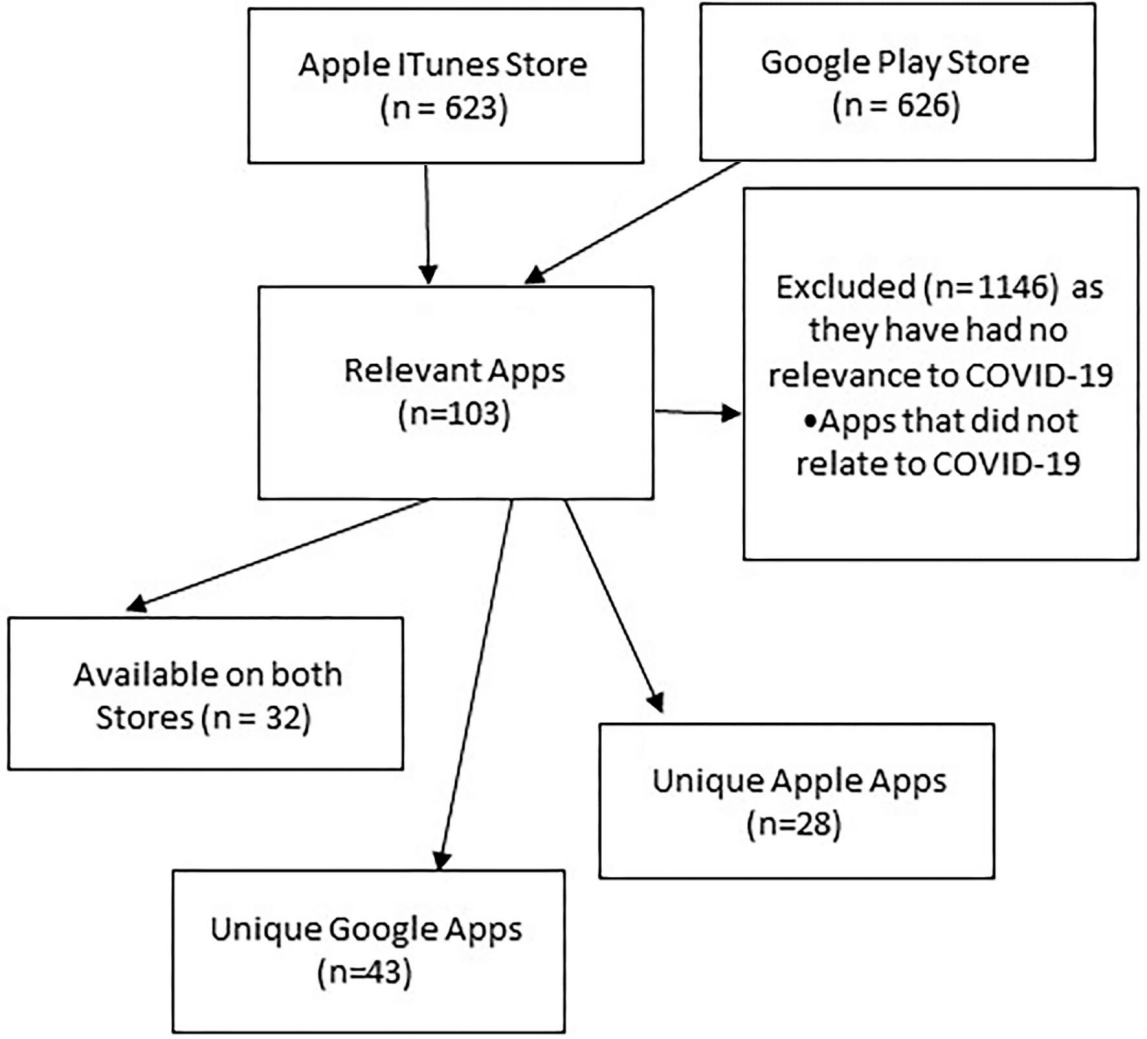
Flowchart demonstrating how applications were identified.

There were 623 applications identified from the Apple iTunes store and 626 applications from the Google Play store. After further screening, as described above by two independent reviewers, there remained a total of 103 applications were identified from the Apple iTunes and Google Play store, respectively; 32 were available on both Apple and Google Play stores. Thus, in terms of unique applications per store, there were a total of 28 from the Apple Store and 43 from the Google Play store. Figure 1 provides an overview of the selection process of the applications. Supplementary Table 1 provides a summary of the core characteristics of the applications from both the commercial app stores. Of the identified applications, 26 applications are from the United States, Canada and Mexico; 29 applications are from the United Kingdom and rest of Europe; 1 application is from Australia and 18 applications are from India and Pakistan. Of the identified applications, at least 13 applications have had inherent contact tracing capabilities; 27 apps have had the main functionality of information provision and 12 apps have had functionalities relating to self-tracking of symptoms. Thirty-eight applications are classified under the “Health and Fitness” category and 39 applications are under the “medical category.”

**Figure 2:**
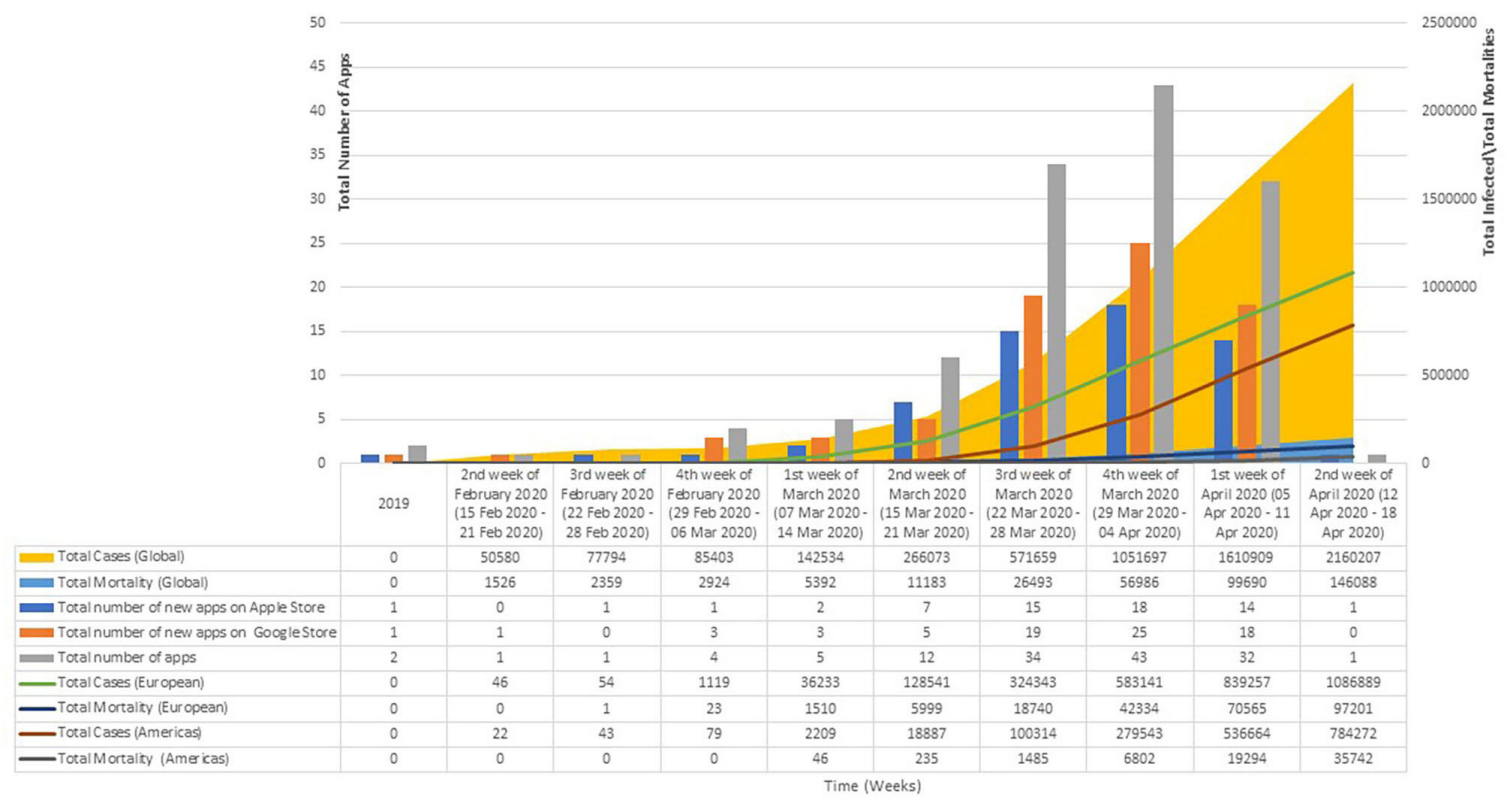
Availability of commercial apps by total numbers of infected/mortalities. Sunday is taken as the 1st day of the week for the computation of the number of new applications that are available on the stores. The numbers of individuals infected, and the total moralities figures have been extracted from the World Health Organization's COVID-19 Situation Reports. There were no available figures for 2019 as the first WHO Situation Report was published on 21 Jan 2020. The line graphs present the overall total number of infections and mortalities in Europe and the United States.

Figure 2 provides an overview of the numbers of new application available on the stores per week, along with an overview of the total numbers of individuals infected and the total number of mortalities.

As evident from Figure 2, there was a steady increase in the total number of applications made available, with most applications being available in the 3rd week of March through to the 2nd week of April. This corresponds to the period on which there is an exponential increase in the numbers of individual infected and the total number of mortalities. A total of 11 applications were made available for individuals living in the United States in March 2020 and at least 22 applications were made available for individuals living in Europe in this time period. Of these applications, at least six of these applications allowed for some form of self-assessment or screening, 11 for the provision of information pertaining to COVID-19 and two with features that enables for contact tracing. Some applications also reported functionalities that allows healthcare professionals to remotely monitor individuals who were discharged from a medical facility following treatment for COVID-19. Amongst the applications made available in the months of April 2020, at least five were made available for individuals living in the United States, and six were available for individuals living in the Europe. Of these applications, at least seven provided tools that allowed individuals to self-assess for symptoms related to COVID-19. Amongst all the identified applications, almost all, but 13 did not include any mention or links to a privacy policy. Almost all of the identified applications have stated the developer or the governmental organizations that were involved in the creation of the applications.

## Discussion

From our knowledge, this is perhaps the first review that has systematically characterized all the applications on COVID-19 from the commercial stores. We found 103 applications that are commercially available for individuals who are concerned about COVID-19. The majority appeared on the commercial stores between March and April 2020, more than 2 months after the first discovery of COVID-19. Most of the applications that were created in these 2 months catered to individuals who were living in countries that were severely affected by COVID-19, that of Europe and the United States. Some of the common functionalities include the provision of news and information, contact tracking and self-assessment or diagnosis. Most of these applications have been jointly conceptualized with governmental organizations, and with the majority having been recently updated.

As evident from our results, there has been a proliferation of applications in the months of March through to April, and this corresponds to the increasing numbers of individuals infected and the mortalities globally. We found there to be an increasing number of applications (*n* = 22 in March for Europe; *n* = 11 in March for United States, *n* = 6 in April for Europe, and *n* = 5 in April for United States) in catering to the needs of the individuals from Europe and the United States, and this corresponds to the rapidly raising numbers in these two regions. As of the 21st of April 2020, the World Health Organization reported the European Region to have the greatest number of cases (1,149,071), and deaths (103,5886) (2) and within these figures, Spain and Italy are ranked the 1st and 2nd (2). As of 21st of April 2020, there were a total of 178,972 cases in Italy, with 23,660 deaths, whereas for Spain, there were a total of 195,944 cases and 20,453 deaths (2). In America, as of the 21st of April 2020, there has been a total of 893,119 infections with a total mortality that of 42,385. The proliferation of these applications might be due to the measures introduced by the different governments, such as a lockdown of the major cities, and these applications thus are integral in the provision of news and information. These applications might also help in the self-assessment of individuals for symptoms of COVID-19, so that individuals would know when and where to seek help. Additionally, as highlighted in the results, some of these applications allow for contact tracking, which is crucial in an epidemic to stem the increasing rates of infection.

Our results show that there is an increase in the numbers of applications only 2 months since the first discovery of COVID-19. Applications have been made available on the commercial stores since the start of the pandemic, but there were concerns pertaining to the information quality and the accuracy of the information within these applications, and this led to both Apple and Google announcing a ban on commercial applications from 5th March 2020 (12). This ban was to reduce the possibility of applications disseminating inaccurate information or fake news, recognizing that there was already a significant degree of anxiety amongst the general public, which has been reported formally in studies (5, 13, 14). Therefore, the applications we identified have attributions to a governmental organization, as this was necessary for it to be featured in a commercial app store. To date, the ban on applications have since been lifted.

From our review, the most common functionality of applications was in the provision of information about the nature of the disease outbreaks. Some applications incorporated novel functionalities, such as the ability to integrate with medical records, or link up with physical equipment like pulse oximeters, or harness the geo-location services in the smartphone for contact tracing. The provision of accurate information in times of a pandemic is crucial, to keep the general public well-informed of the changes in the situation and allay public's anxieties. The importance of accurate information has been echoed by the Centers for Disease Control and Prevention (CDC); they recommended guidelines to help public health authorities work with news media agencies, to ensure that the information disseminated is accurate (15). The fact that these applications allow for integration with physical sensors makes it possible to track patients remotely, such as those who have recently recovered. This helps to ensure that these patients could still be monitoring for residual symptoms despite them not being in a medical facility. A recent evaluation of a COVID-19 remote patient monitoring applications [Annis et al. (16)] (GetWellLoop) tested amongst 2,255 participants found that it was effective in enabling patients to manage their COVID-19 symptoms at home. Lastly, the functionality that allows for tracking of the users ought to be replicated for other countries, as it might be an inexpensive method for contract tracking.

At the time of the conclusion of our search, we found no applications that could assist in the delivery of psychological intervention, or counseling support are non-existent. These applications could be beneficial for patients afflicted with COVID-19 and members of the general public, who might require support given their high levels of psychological distress. Several recent studies have documented high levels of psychological distress amongst the general public. For example, Wang et al. (17) investigated, using the IES-R and the Depression, Anxiety and Stress Scale (DASS-21), the mental health status of 1,120 members of the general public living in China. Notably, 53.8% reported the psychological impact to be moderate to severe, with 16.5% having moderate to severe depressive symptoms, 28.8% moderate to severe anxiety symptoms and 8.1% moderate to severe stress. Liu et al. (5) have highlighted the high levels of psychological distress and the way in which apps have been used in China to help support these individuals together with social media networks, such as WeChat, to deliver of psychological interventions (5). It is thus crucial for clinicians to work with software developers in joint conceptualization of the next generation of apps for COVID-19 that could cater to the psychological needs of the end users. It is important to include clinicians, as they might be able to provide resources for the evidence-base of these apps. It might also be ideal to have patient participants to share ideas and offer their perspectives as to what they need from an application, to ensure that the apps meet the specific needs of users. By the time of revision of this manuscript in December 2020, we are now made aware that there have been applications, such as Corona Health (18), that attempts to investigate the impact that COVID-19 has on the mental health of adolescents and adults. Unfortunately, this application was only created several months into the pandemic. Such tools might be extremely helpful for the examination of the acute psychological distress of the public during the initial periods of the pandemic.

One of the major strengths of this study is that we have identified all the commercial COVID-19 applications featured on the two most common application stores. These commercial applications are likely to be resources that the general public would turn to, in such times. By using a global search engine, we were able to identify applications across the regions, instead of a manual search, which would confine our results to a particular locality, and would not have provided an overview of the nature of the COVID-19 app landscape. However, our study is not without its limitations. We have searched for COVID-19 applications using a search engine by means of keywords search and will have had missed out any applications that do not use the term COVID-19 in their application names, unfortunately this is a limitation that we were unable to overcome with the existing search tools that used. Our comprehensive searched of the two largest commercial stores did not identify any applications developed in China, and yet we know from Liu et al.'s (5) review that such apps exist. This is possibly because China-developed applications are not routinely hosted on these two commercial application stores, and are hosted on China application stores, which are not searchable outside of China. We have not tested the functionalities of the apps identified, as we were limited by resource and linguistic skills, also some applications require the users to register with credentials from that country. In addition, we are also aware that there have been tools that allow for the analysis of user reviews of applications. Such tools might provide a better perspective into the usefulness of each of the identified application and the challenges that individuals face in using them. We have not considered the use of such tools in our current work, as our intents were in characterizing the type and functionalities of applications that were made available in the early days of the pandemic. This is a dynamic field and we have summarized the state of play within 3 weeks, it will be interesting to observe whether new apps are launched with the same frequency and the focus of their content. Nevertheless, understanding the landscape of commercial applications available can inform the preparation of health promoting materials and highlight to developers where need still remains.

## Conclusion

This is the first review to characterize the smartphone applications which are currently available, 4 months after the first discovery of COVID-19. Whilst there are several diverse applications mainly for the provision of information and tracking of health status, there remains a need for applications that could address the psychological well-being of the general public. The information generated by this review will also inform health professionals and the general public about the applications they can recommend or use for emotional support during the COVID-19 outbreak and it is also of importance for governmental policy and planning, as some of these technologies, if scientifically validated to be effective, could be shared amongst countries.

## Data Availability Statement

The raw data supporting the conclusions of this article will be made available by the authors, without undue reservation.

## Author Contributions

MZ, AC, RH, and HS jointly conceptualized the study. MZ and AC were involved in data extraction, verification of the extracted data, and amended the second draft of the manuscript. MZ worked on the first draft of the manuscript, which HS provided guidance and further amends. RH provided critical updates to the final manuscript. All authors read and approved the manuscript prior to submission.

## Conflict of Interest

The authors declare that the research was conducted in the absence of any commercial or financial relationships that could be construed as a potential conflict of interest.
